# Effectiveness of Virchow’s Node Fine-Needle Biopsy for the Discovery of Occult Metastatic Prostate Cancer

**DOI:** 10.7759/cureus.27795

**Published:** 2022-08-08

**Authors:** Luciana Izquierdo Del Barco, Guillermo Izquierdo Pretel, Marco Ruiz

**Affiliations:** 1 Internal Medicine, Florida International University, Miami, USA; 2 Internal Medicine, Florida International University, Herbert Wertheim College of Medicine, Miami, USA; 3 Miami Cancer Institute, Florida International University, Herbert Wertheim College of Medicine, Miami, USA

**Keywords:** prostate cancer, fna biopsy, metastatic prostate cancer, virchow's node, fine-needle aspiration

## Abstract

In this case report, we describe the effectiveness of fine-needle aspiration of Virchow’s node for the diagnosis of metastatic prostate cancer in a 62-year-old male without any medical history, negative urinary tract symptoms, and a normal digital rectal examination. The patient presented with respiratory distress, diffuse lymphadenopathy, and high levels of prostate-specific antigen. Multiple studies were done with inconclusive results until positive findings of the *NKX3.1* gene were found in the immunostain smear of the Virchow’s node, which led to the identification of metastatic prostate cancer.

## Introduction

Prostate cancer is the second most common cancer and the second leading cause of cancer-related death in American men [1]. Prostate cancer can be diagnosed by lower urinary tract symptoms and/or with appropriate screening. These include digital rectal examination (DRE) and prostate-specific antigen (PSA) in the adult and elderly population. The diagnosis of advanced prostate cancer can be challenging, especially in cases where the patient does not have symptoms and has a normal rectal examination. It is also challenging as other types of cancer can have similar presentations. Here, we present the case of a 62-year-old Hispanic male with cough and shortness of breath along with diffuse lymphadenopathy and marked elevation of PSA.

While different examinations alerted the primary care team of different etiologies of cancer, they had begun to rule out that the primary cancer was from the prostate because the clinical examinations, including rectal examination and transrectal ultrasound, were deemed normal despite alarmingly high PSA levels (402 ng/mL) throughout the patient’s stay in the hospital.

PSA is a protein produced by the epithelial cells of the prostate gland. It serves to liquefy the semen in the seminal coagulum allowing the sperm to move freely. PSA is present in the serum in small quantities in healthy people but is elevated in case of prostate cancer or other prostatic disorders [2]. In our case, it was only after the fine-needle biopsy of the patient’s Virchow’s node that clarified any hypothesis surrounding the diagnosis of metastatic prostate cancer.

On the primary examination, the patient presented with a firm, non-tender lump in the left supraclavicular region between the two heads of the sternocleidomastoid muscle (Virchow’s node). The patient did not have any other palpable nodes. Virchow’s node correlates with metastatic cancer in the gastric regions, and, in rare cases, it can be present because of prostate cancer [3]. Thus, this case highlights the importance of considering prostate cancer in the differential diagnosis of male patients presenting with Virchow’s node and performing a fine-needle aspiration (FNA) biopsy to confirm the diagnosis.

FNA is a diagnostic procedure used to investigate lumps or masses. This technique is performed by inserting a thin hollow needle into the lump and collecting its cells which are then stained to be examined under the microscope. The FNA biopsy is a very safe minor surgical procedure that can be done by the palpation of the mass, or with the assistance of images such as ultrasound or computed tomography (CT) scan. Puncturing tissues through a fine needle is a diagnostic technique that has developed mainly in the last century and is accepted globally [4]. In conjunction, the development of cytological techniques has been crucial in achieving the effectiveness of diagnosis.

## Case presentation

A 62-year-old male presented to the emergency department complaining of progressive dyspnea and dry cough for two months. Two weeks before the emergency admittance, the patient was admitted to a peripheral facility for the same symptoms and had been clinically diagnosed with viral vs. bacterial pneumonia. He was treated with antibiotics and discharged home, but the symptoms persisted. After several visits to his primary provider who ordered a positron emission tomography-computed tomography (PET-CT) skull base to mid-thigh scan, there was mass-like substantial fluorodeoxyglucose (FDG) uptake in the proximal sigmoid colon, and numerous FDG-avid lymph nodes were seen in the abdomen, pelvic, and left the supraclavicular area, along with scattered ground-glass and interstitial opacities in the lungs.

The patient arrived at the emergency room (ER) with dyspnea and oxygen saturation of 87% at room air. No previous relevant medical history was noted. On initial examination, labs were unremarkable except for arterial blood gas (ABG) analysis with low PaO_2_, elevated D dimer, and PSA of 402 ng/mL. The viral panel including coronavirus disease 2019 was negative. He was also found with abnormal chest X-ray (CXR) and CT chest findings with diffuse ground-glass opacities on both upper lungs and multiple lymphadenopathies. No evidence of segmental pulmonary embolism was noted. There were no lower urinary symptoms and no previous colonoscopy. The patient reported no marked decrease in weight and no fever.

**Figure 1:**
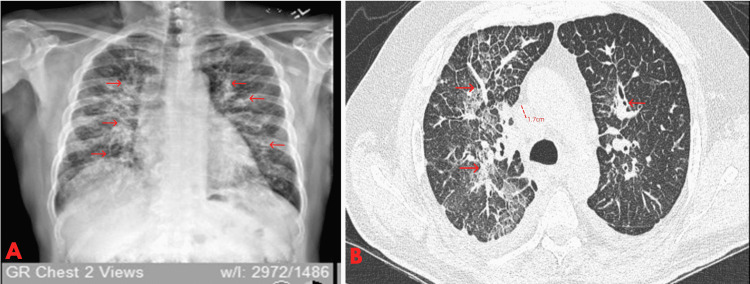
Chest X-ray and CT findings. A. Chest X-ray denoting diffuse bilateral patchy/heterogeneous opacities with prominent interstitial markings. B. Chest CT showing severe diffuse bilateral septal line thickening with patchy ground-glass opacity. Diffuse severe peribronchial thickening. Multifocal thoracic lymphadenopathy measuring up to 1.7 cm in size.

Figure 1A shows the patient’s CXR depicting diffuse bilateral opacities with notable interstitial markings. Figure 1B shows the patient’s CT scan. As shown in the images in red, the patient had multifocal thoracic lymphadenopathy measuring up to 1.7 cm in size. On the CT scan, there were marked opacities throughout the parenchyma, along with the presence of severe peribronchial thickening.

The patient was a Cuban immigrant who had lived in Miami for over 10 years. He used to work in a metallic factory many years ago. The patient was married and lived with his family. He had never smoked and sporadically consumed alcohol at social gatherings. He denied the use of illicit drugs, and there was no family history of cancer. He was not taking any medications.

On physical examination, he was awake, alert, and oriented to person, place, or time. He was not in acute distress and had good nutrition. Examination of the head, eyes, ears, nose, and throat was unremarkable. Small hard nodes were palpable on the left supraclavicular area. Bilateral basal crackles were heard on both lungs, but no clubbing, cyanosis, or edema was present. The cardiovascular examination was normal, and his abdomen was normal. On rectal examination, the size of the prostate was normal, and no induration or nodules were present. Hemogram was normal, and no rheumatologic common positive markers were noted. Erythrocyte sedimentation rate and C-reactive protein were not elevated. Blood cultures were also negative.

Upon CT examination of the abdomen/pelvis, extensive retroperitoneal lymphadenopathy, with nodal conglomerates at the level of the kidneys was observed. At the same level, periceliac adenopathy, aortoiliac bifurcation adenopathy, left internal iliac, and left obturator adenopathy were also present.

A conglomerate of enlarged hypoechoic lymphadenopathy measuring approximately 3.2 cm in the left supraclavicular region was observed with the help of a linear ultrasound.

Consideration of metastatic cancer was acknowledged, and colonoscopy, bronchoscopy, and fine-needle biopsy from the left supraclavicular node were requested. Bronchoscopy findings were not relevant, thus the epiglottis, vallecula, and the right and left pyriform sinuses, as well as the arytenoids, were deemed normal-appearing. Additionally, no endobronchial lesions were noted on bronchoscopy. Results from bronchoalveolar lavage were also negative for infection and malignancy.

The gastroenterology team completed a colonoscopy, and several polyps and a non-obstructive sigmoid mass were observed and biopsied. Carcinoembryonic antigen was negative. Biopsy was negative for malignancy.

**Figure 2:**
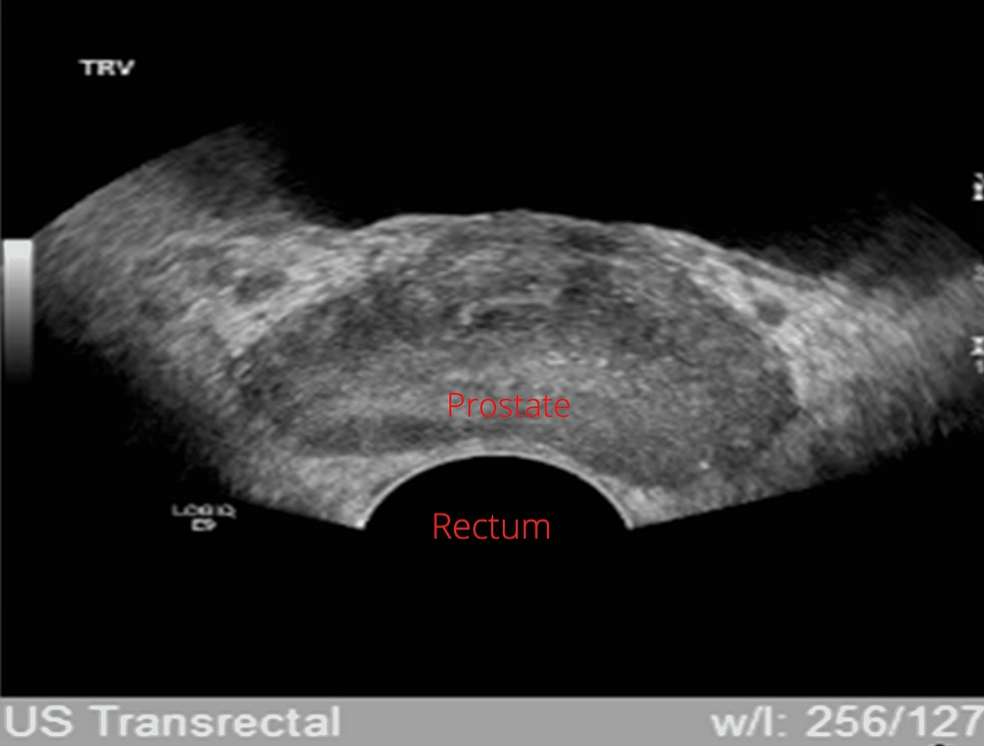
The prostate measures 4.5 × 3.5 × 2.5 cm with an estimated volume of 21 mL. There is normal echogenicity and vascularity. A sub-centimeter cyst is seen in the periphery of the posterior mid-gland/base. A 0.6 × 0.5 cm hypoechoic nodule is seen in the right medial transition zone.

Figure 2 depicts the patient’s prostate. Several tests were conducted, including a transrectal ultrasound to identify the root of the patient’s disease. Due to the isolated and exceedingly elevated PSA levels, urology was consulted. The transrectal ultrasound was completed and a small sub-centimeter nodule was observed.

Urology did not proceed with prostate biopsy as the patient was positive for malignancy in the FNA. Immunostaining was positive for *NKX3.1*, consistent with carcinoma of prostate origin in the FNA biopsy from the left supraclavicular node.

**Figure 3:**
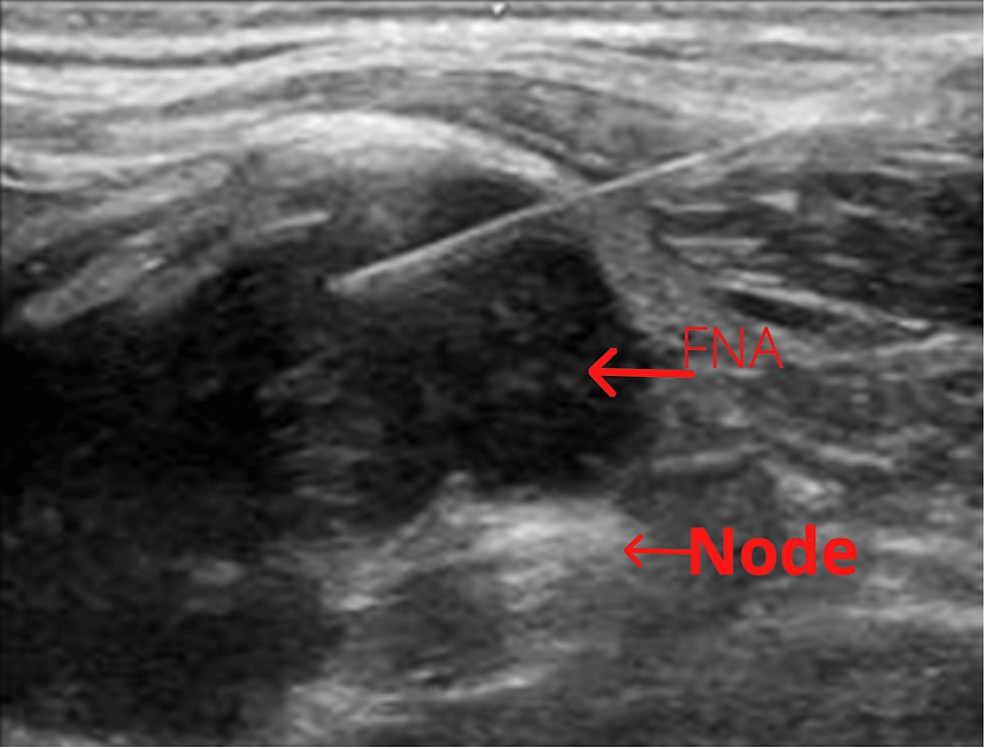
Ultrasound-guided fine-needle aspiration of the left supraclavicular lymph node.

FNA and ThinPrep and smears were performed for the left supraclavicular lymph node (Virchow’s node), which were markedly positive for malignancy (Figure 3). Immunocytochemistry performed on a previously Papanicolaou-stained smear showed that the tumor cells were positive for *NKX3.1*, consistent with carcinoma of prostate origin.

Treatment for the newly diagnosed prostate cancer and elevated PSA was initiated with oral bicalutamide 50 mg daily for two weeks before administering outpatient androgen deprivation therapy (ADT) to prevent an androgen storm. The patient was then started on ADT with leuprolide 22.5 mg injections every three months plus docetaxel (peripherally) every three weeks for six courses.

Differential diagnosis

Metastatic colon cancer was considered in the differential due to positive PET-CT scan findings. In addition, sarcoidosis of the prostate was mentioned as a rare possibility considering diffuse lymphadenopathy and because it could have elevated PSA levels. 

Laboratory findings

Table 1 details the patient’s laboratory findings at the time of admission, as well as the normal reference values for consideration. Note the elevated levels of PSA at 402.3 ng/mL (normal range: 0.0-4.0 ng/mL).

**Table 1:** Laboratory results at the time of admission.

Laboratory	Admission time results	Reference values
Arterial pH	7.45	7.35–7.45
Arterial carbon dioxide	33 mmHg	35–45 mmHg
Oxygen arterial pressure	53 mmHg	75–100 mmHg
Arterial bicarbonate	22 mmol/L	19–24 mmol/L
Arterial oxygen saturation	87.6%	Over 92%
White blood cell count	9.9 × 10^3^/µL	4.0–10.5 × 10^3^/µL
Red blood cell count	5.40 × 10^6^/µL	4.2 – 5.6 × 10^6^/µL
Hemoglobin	14.9 g/dL	13.3–16.3 g/dL
Hematocrit	46.0%	39.0–47.1 %
Platelet count	190 × 10^3^/µL	140–400 × 10^3^/µL
D dimer quantitation	8.93 µg/mL	0.00–0.49 µg/mL
Whole blood glucose	73 mg/dL	74–106 mg/dL
Whole blood sodium	139 mmol/L	137–145 mmol/L
Whole blood potassium	3.8 mmol/L	3.6–5.0 mmol/L
Blood urea nitrogen	10 mg/dL	9–20 mg/dL
Creatinine	0.71 mg/dL	9.66–1.25 mg/dL
Calcium	8.7 mg/dL	8.4–10.2 mg/dL
Total protein	6.3 g/dL	603–8.2 g/dL
Albumin	3.1 g/dL	3.9–5.0 g/dL
Prostatic-specific antigen	402.3 ng/ml	0.0–4.0 ng/ml
Aspartate aminotransferase	25 U/L	15–46 U/L
Alanine transaminase	13 U/L	21–72 U/L
Lactate dehydrogenase	606 U/L	313–618 U/L
C-reactive protein	1.6 mg/dL	0.0–0.9 mg/dL

Treatment

After confirming the diagnosis on FNA biopsy, treatment was initiated for the patient with oral bicalutamide 50 mg daily for two weeks. After being released from the hospital he was on ADT to prevent androgen storm with leuprolide 22.5 mg injections every three months plus docetaxel (peripherally) every three weeks for six cycles.

Outcome and follow-up

After three weeks of the initial treatment, the patient noted less dyspnea and his oxygen saturation improved. His performance status returned to normal. After five months of treatment, his PSA was 2.6 g/mL. He was followed for 16 months with marked improvement, and a normal PSA was noted. After 19 months of treatment, the patient is grateful for the treatment; he considers himself a survivor and is following treatment diligently. He has expressed gratitude toward his primary care team and is hopeful for the future. It has to be noted that his mood has always been cheerful throughout the treatment, and he remains in good spirit.

## Discussion

The diagnosis of metastatic prostate cancer is a challenge due to the similar presentation with cancers originating from other organs, including the gastrointestinal tract, pancreas, or biliary tract, as well as the liver or the lungs. To guide interventions, a logical clinical diagnosis is needed to establish the most appropriate workup. These logical statements could be guided by epidemiology including the answer to the question “what are the most common primary cancers in patients with Virchow’s node?” In association with the possible answer, we must consider the results of the puncturing with FNA and cytology which can give us the most effective method to reach the diagnosis.

Virchow’s node, a left supraclavicular lymph node, was first described by German pathologist Rudolf Virchow in 1848, as a sign of metastatic malignancy, mainly from gastric cancer. The Troisier’s sign is used interchangeably with Virchow’s node. The mechanism of this lymphadenopathy is due to tumor embolization from the primary sites through the thoracic duct, which eventually involves Virchow’s node and the cancer cells become trapped and become enlarged. Several studies have established its association with various malignancies, including gastrointestinal, pulmonary adenocarcinoma, prostate cancer, and lymphoma, among others. The initial presentation of a prostate adenocarcinoma as a left supraclavicular mass is unusual. The appearance of a left supraclavicular mass is not generally related to prostate cancer because its incidence is very low, being reported between 0.4% and 1% of all cases of metastatic prostate cancer [5]. It is also positive in certain infections such as tuberculosis and syphilis [6-8].

The case presented has shown highly significant levels of PSA, as clearly stated in the laboratory data, and this progress has been complemented by the utility of immunostaining in the detection of metastatic prostate cancer [8]. It has helped in the detection of the *NKX3.1* gene, which is a superior indicator of metastatic prostate cancer compared to the PSA levels and the prostatic acid phosphatase (PAP) on cell smears. It is superior because the *NKX3.1* is a nuclear maker while the PSA and the PAP are cytoplasmic markers, making the latter less reliable for the identification of metastatic prostate cancer.

## Conclusions

If Virchow’s node is present, FNA is the most effective tool to determine the etiology of metastatic cancer. We must exercise logical reasoning to establish a clinical diagnosis that limits additional examination and extensive workup done on the patients. If metastatic prostate cancer is suspected, immunostaining to detect the *NKX3.1* homeobox gene should be requested. A good physical examination is imperative to determine the presence of nodes. With the ever-evolving medical technology, the easy accessibility of ultrasound can be used to identify nodes.
